# The Investigation of HBV Pre-S/S Gene Mutations in Occult HBV Infected Blood Donors with anti-HBs Positive

**DOI:** 10.1155/2022/1874435

**Published:** 2022-07-19

**Authors:** Yan Guo, Yu Lan, Yuanyuan Jing, Bin Cai, Hanshi Gong, Yixin Zhang, Yong Duan

**Affiliations:** ^1^Clinical Laboratory, Shaanxi Blood Center, Zhuque Street No.407, Yanta District, Xi'an, Shaanxi 710061, China; ^2^NHC Key Laboratory of Biosafety, National Institute for Viral Disease Control and Prevention, Chinese Center for Disease Control and Prevention, ChangBai Road No.155, Changping District, Beijing 102206, China

## Abstract

**Introduction:**

The coexistence of hepatitis B virus (HBV) and hepatitis B surface antibodies (anti-HBs) in occult hepatitis B virus infection (OBI) is a contradictory phenomenon, and the underlying mechanism is not fully understood. The characteristics of pre-S/S mutations in OBI genotypes B and C (OBI_B_ and OBI_C_) in the presence or absence of anti-HBs were analyzed extensively in this study. *Methodology*. The amino acid substitutions of envelope proteins of 21 OBI strains, including 4 HBs (+) OBI_B_, 6 HBs (−) OBI_B_, 6 HBs (+) OBIc, and 5 HBs (−) OBI_C_ samples, were analyzed and fully compared among groups of HBV genotypes and the presence of anti-HBs.

**Results:**

The mutation rates in pre-S1, pre-S2, and S proteins of OBI_C_ were significantly higher than wild-type HBV (wt-HBV) genotype C strains, but only the mutation rate of S protein in OBI_B_ was significantly higher compared to wild-type HBV genotype B. The mutation rates in S protein of anti-HBs (−) OBI were higher than anti-HBs(+) OBI samples (4.40% vs. 2.43% in genotype B, *P* > 0.05; 6.81% vs. 3.47% in genotype C, *P* < 0.05). For these high-frequency substitutions in the pre-S/S region, the mutations sN40S and sK122R were found in 27.3% and 45.5% of anti-HBs (−) OBI strains, respectively. 7 substitutions were uniquely found in OBI_C_ strains and 9 substitutions were commonly detected in OBI_B_ and OBI_C_ strains.

**Conclusions:**

These results suggested that the mutations might occur randomly and were not selected by antibody pressure.

## 1. Introduction

Hepatitis B virus (HBV) infection is a major threat to human health worldwide, and nearly 2.57 billion people worldwide are estimated to be infected with HBV. China is a higher intermediate prevalence area of HBV. The prevalence of HBsAg for populations aged 1∼59 years is 7.18%. It is estimated that there are more than 93 million HBV-infected individuals in China, which results in a public health issue [1]. Occult hepatitis B virus infection (OBI) is a special type of hepatitis B virus (HBV) infection, which is characterized by the presence of a low viral load in the liver and/or in the blood of individuals and being negative for HBV surface antigen (HBsAg) [2]. OBI could be transmitted through blood, and the minimal HBV DNA infectious dose is below the detection limit of the current nucleic acid amplification technology assays (NAT) [3], so a residual risk of transfusion-transmitted OBI still exists in qualified blood donors even after the routine serological and nucleic acid screening, which results in a threat to the safety of blood transfusions and poses extra challenges to the prevention and control of HBV infection.

OBI can be grouped into two types: seropositive OBI [hepatitis B core antibody (anti-HBc) and/or anti-hepatitis B surface antibody (anti-HBs) positive] and seronegative OBI (anti-HBc and anti-HBs negative) [2]. The anti-HBs antibody is usually considered a marker of successful virus clearance and long-term protection. However, the coexistence of HBV and anti-HBs is quite common in OBI [4, 5]. Mu et al. reported that the prevalence of occult HBV infection was 10.9% in HBV vaccinated children in Taiwan [6]. 124 of 2919 (4.2%) HBV vaccinators at the age of 19∼21 were found to be HBsAg (−), anti-HBs (+), and anti-HBc (+), in which HBV DNA was detectable in 81 sera samples using nested PCR [7]. Pande C's investigation results were even more surprising [8]. In 213 babies born to HBsAg (+) mothers who received recombinant HBV vaccinations at 0, 6, 10, and 14 weeks, 9/213 (4%) developed overt HBV infection, and 89/213 (42%) developed occult HBV infection at a median of 24 months of age. It is worth noting that 51% (45/89) of OBI babies received hepatitis B immunoglobulin (HBIG) and recombinant HBV vaccine, suggesting that OBI in infants with HBV-infected mothers might not be prevented by HBV vaccination.

In general, the level of anti-HBs is low in OBI patients; for example, 4 of 120 HBsAg(−) healthcare workers with low (<10 IU/L) and moderate levels (>10 to <100 IU/L) of anti-HBs were positive for HBV DNA examined by sensitive real-time PCR [9]. It seems that low and moderate levels of anti-HBs have limited neutralization capacity to prevent OBI completely, but a high level (>100 IU/L) of anti-HBs could not provide full protection either, as reported by Zheng et al. [10].

The mechanisms behind the occurrence of OBI associated with anti-HBs pressure are not fully understood. In this study, the pre-S/S mutations in OBI blood donors associated with anti-HBs elicited by vaccination or the host immune response to HBV were analyzed extensively, providing new data for the coexistence of HBV anti-HBs and even the mechanisms of OBI.

## 2. Methodology

### 2.1. Blood Sample Collection and Screening

136425 blood samples were collected by the Shaanxi Blood Center from January to October 2015. Blood samples were individually screened for anti-HIV, anti-HCV, HBsAg, and anti-TP by enzyme immunoassays (EIA) using two different reagents for two rounds (kits were from Wantai Biological Pharmacy Enterprise Co. Ltd., Zhuhai Livzon Diagnostics Inc., Shanghai Kehua Bio-engineering Co. Ltd., Italy's Diasorin Company and USA Bio-Rad Company). The level of alanine aminotransferase (ALT) was detected by the rate method (Beckman Coulter Au Chemistry Systems, USA).

HCV, HIV, and HBV nucleic acids were detected in 6 samples mixed-mode (6×167 *µ*L) of the Roche COBAS S 201 Nucleic Acid Detection System (F. Hoffmann La Roche Ltd., Basel, Switzerland). The reactive pools were identified according to the single sample mode. All HBsAg (−)/HBV DNA (+) samples were confirmed and quantified by electrochemiluminescence immunoassay (ECLIA) using a reagent kit from Wantai (Wantai Biological Pharmacy Enterprise Co. Ltd.). The study protocol was approved by the Ethics Committee of the Shaanxi Blood Center.

### 2.2. HBV DNA Quantification and Amplification

Real-time fluorescence-based quantitative PCR was performed to measure HBV DNA levels. Sample handling procedures were strictly according to the instructions supplied by the reagent kit (Sansure Biotechnology, Hunan, China). The minimal detection limit for HBV DNA is 10 IU/mL, and the linear range of the standard curve is from 20 IU/mL to 2.0 × 10^9^ IU/mL.

The Pre-S/S (1434 bp)/S (480 bp) region of the HBV genome was amplified by nested PCR according to a previous study [11]. HBV DNA was extracted from plasma using TIANamp Viral DNA/RNA kits (Sansure Biotechnology, Hunan, China). The two sets of PCR primers for the PreS-S gene were:

P1:5′-ACATACTCTTTGGAAGGCKG-3′; R1:5′-CGTCAGCAAACACTTGGC3′; P2:5′GCCTCATTTTG

YGGGTCA-3′; R2:5′-AGCAAARCCCAAAAGACC-3′. The two sets of PCR primers for the S gene were: S1:5′-CTCGTGTTACAGGCGGGGTTTTTC-3′; R1:5′-CATCATCCATATAGCTGAAAGCCAA ACA-3′; S2:5′-TTGTTGACAAGAATCCTCACAATACC-3′; R2:5′-GCCCTACGAACCACTGAACAA ATGG-3′. The products were purified and sequenced by Jinweizhi Biotechnology Co., Ltd. using ABI 3730 DNA sequencer (Applied Biosystems).

### 2.3. Sequence Analysis of HBV DNA

In the Kimura-2-parameter model with 1000 bootstrap replicates, phylogenetic trees were constructed using Mega-X software using the neighbor-joining method. The HBV reference sequences for multiple genotypes (A to H) were obtained from the National Center for Biotechnology Information genotyping tool. The deduced amino acid sequences of the pre-S/S regions (pre-S1, pre-S2, and S) of OBI were compared to the corresponding genotype B or C consensus sequences of HBV wild-type strains. The control HBV wild-type strains were all Chinese strains obtained from the GenBank database. Accession number of genotype B were FJ386582, FJ386600, FJ386608, FJ386610, FJ386634, FJ386654, FJ386655, FJ386658, FJ386668, FJ386669, FJ386680, FJ386681, FJ386683, FJ386684, FJ386688; accession number of genotype C were FJ386577, FJ386579, FJ386585, FJ386587, FJ386603, FJ386604, FJ386614, FJ386619, FJ386639, FJ386644, FJ386649, FJ386657, FJ386661, FJ386662, FJ386685.

### 2.4. Statistical Analysis

The SPSS software 18.0 (SPSS, Chicago, IL, USA) was employed for statistical analysis. Group results were compared using a *t*-test or the chi-square test, as appropriate. A difference with *P* < 0.05 (bidirectional) was statistically significant.

## 3. Results

### 3.1. Serological Characteristics of OBI in Blood Donors

Of the 136425 sera samples, 95 were verified as HBsAg (−)/HBV DNA (+), accounting for 0.0696% of the investigated blood donors. These samples can be classified into 6 different groups based on the serological index (Table 1). 10 samples were seromarker negative, indicating that those samples were from blood donors in a window period of infection or with seronegative/primary OBI. They were removed for further study due to their uncertainty. The other 85 samples were defined as OBI and used in the following study. Of the 85 OBI samples, 34 were detected as anti-HBs positive, including 7 samples with anti-HBs concentrations of over 100 IU/L, 15 samples with anti-HBs concentrations between 10 IU/L and 100 IU/L, and 12 samples with anti-HBs concentrations lower than 10 IU/L. The other 51 samples were anti-HBs negative. All the samples had normal ALT levels (<40 IU/L). The average age, the ratio of women to men, and the viral load in the anti-HBs (+) and anti-HBs (−) donors were 40.71 vs. 43.82; 9 : 25 vs. 9 : 42; 47.76 ± 28.91 IU/mL vs. 21.86 ± 10.43 IU/mL, respectively.

### 3.2. The Amplification of HBV Pre-S/S Gene and Classification of HBV Genotypes

The pre-S/S region of HBV was amplified in 11 samples, including 8 anti-HBs (+) and 3 anti-HBs (−).

The S region was amplified in those samples that failed to detect the pre-S/S region, and 10 samples, including 2 anti-HBs (+) and 8 anti-HBs (−), were amplified successfully. Finally, the pre-S/S genetic regions were successfully amplified in 21 of 85 OBI samples (24.71%). HBV genotypes were classified.

**Figure 1:**
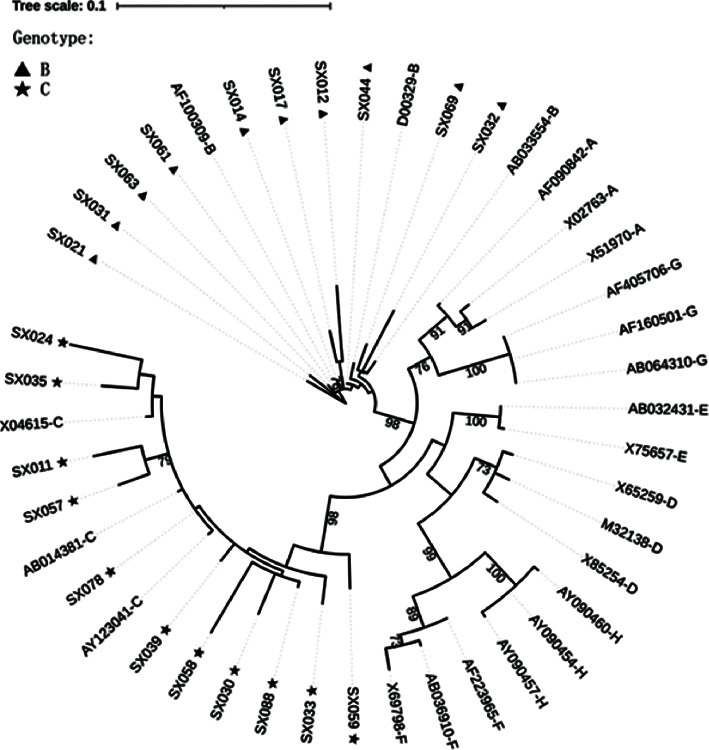
Neighbor-joining phylogenetic tree analysis of OBI S genetic sequences. Analyses were based on 21 S region sequences from blood donors with OBI. Arabic numerals were suffixed with SX represented samples from OBI blood donors; others were reference sequences for multiple genotypes (A through H) from GenBank.

Based on phylogenetic analyses of OBI S region sequences. 10 and 11 strains were classified as OBI_B_ and OBI_C_, respectively (Figure 1). The ratio of females to males was similar for OBI_B_ and OBI_C_ (2 : 8 vs. 2 : 9). The age, ALT levels, and viral load were various between genotypes B and C, but no significant differences were found between the two genotypes (*P* = 0.41–0.85).

### 3.3. Comparison of the Mutations in the Pre-S/S Region of HBV Strains from OBI Samples with that in Wt-HBV Strains

Deduced amino acid sequences of envelope proteins obtained from OBI samples, including 11 pre-S/S and 10 S region sequences from 10 OBI_B_ and 11 OBI_C_ strains, were aligned with the corresponding consensus sequences of wt-HBV strains (15 genotype B and 15 genotype C), and the results of the comparison were presented (Table 2). Compared with genotype C wt-HBV strains, the mutation rates in pre-S1, pre-S2, and S proteins of OBI_C_ were significantly higher (*P* < 0.01). However, the pre-S1 and pre-S2 regions appeared well conserved between OBI_B_ and genotype B wt-HBV strains (*P*=1.000, *P*=0.095), only the mutation rate in S proteins of OBI_B_ was significantly higher (*P* < 0.01) than that of wt-HBV strains. When comparing the sequences of OBIc with OBI_B_, the substitution rate in the pre-S1 region was significantly higher in OBI_C_ than in OBI_B_ (*P* < 0.05), but the substitution rates in pre-S2 and S showed no statistical significance (*P*=0.527, *P*=0.073).

### 3.4. Comparison of the Mutations in the Pre-S/S Region of HBV Strains from OBI Carriers in Groups of anti-HBs Positive and Negative

**Figure 2:**
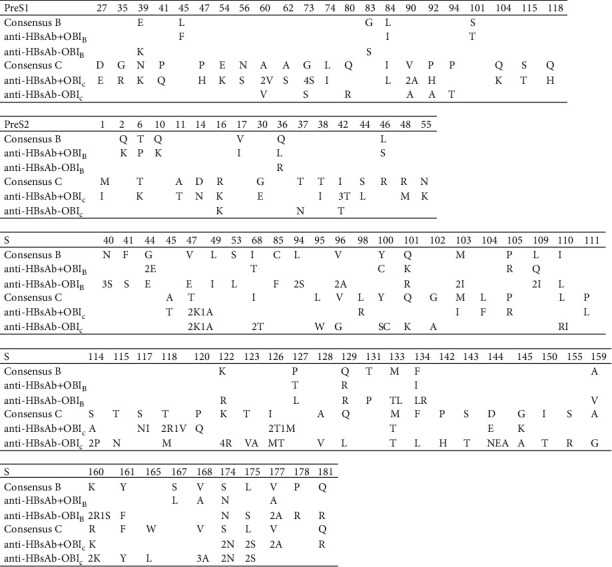
The alignment of amino acid substitutions in the pre-S/S region of anti-HBs (+) and anti-HBs (−) OBI sequences. The Arabic prefix indicated the number of substitutions at each site in OBI sequences. OBI, occult hepatitis B virus infection; anti-HBs, hepatitis B surface antibody.

Site mutations in the pre-S/S region of HBV strains from OBI carriers in the presence or absence of anti-HBs were aligned together (Figure 2). The differences in numbers, frequency, and rates of mutations were evaluated among groups (Table 3). The mutation rate in S protein of anti-HBs (−) OBI_C_ samples was statistically higher than that in anti-HBs (+) OBI_C_ samples (6.81% vs. 3.47%, *P* < 0.05), but no statistical difference was found in anti-HB (−) OBI_B_ and anti-HB (+) OBI_B_ samples (4.40% vs. 2.43%, *P* > 0.05). The substitution rate in pre-S1 and pre-S2 proteins between groups of anti-HBs (−) and anti-HBs (+) in either OBIc or OBI_B_ carriers appeared with no statistical significance (*P* > 0.05). The overall mutation rate considering Pre-S/S in OBIc strains was higher than in OBI_B_ strains. However, for anti-HBs (−) OBI strains, only the mutation rate in S protein presented a statistical difference (*P*=0.046) between genotype B and C, whereas only the mutation rate in pre-S1 protein presented a statistical difference for anti-HBs (+) OBI strains between genotype B and C (*P*=0.01).

Furthermore, amino acid substitutions in the major hydrophilic region (MHR) and the “*α*” determinant were analyzed. The mutation rate of MHR in anti-HBs (−) OBIc strains was significantly higher than that in anti-HBs (+) OBIc strains (10.3% vs. 4.05%, *P* < 0.01), and the mutation rate of MHR in anti-HBs (−) OBI_C_ strains was higher than that in anti-HBs (−) OBI_B_ strains (10.3% vs. 4.52%, *P* < 0.01). Nevertheless, the mutation rates in the “*α*” determinant among different anti-HBs status and genotypes presented no statistical difference.

The high frequency of pre-S/S substitutions was classified as preferentially associated with either the anti-HBs (+) or anti-HBs (−) OBI strains considering different genotypes (Table 4). The substitution sN40S was uniquely found in 27.3% of anti-HBs (−) OBI_B_ carriers, and sK122R was uniquely found in 45.5% of anti-HBs (−) OBI preferentially in OBI_C_ strains. The substitutions ps1A60V, ps1G73S, ps1V90A, ps2I42T, sT118R/V/M, sI126T/M, and sD144E/N/A were uniquely found in OBI_C_ strains in both anti-HBs (+) and anti-HBs (−) populations. The substitutions sV/T47K/A/E, sM133T/L, sF134I/L/R, sK160R/S/K, sV168A, sS174N, sL175S, and sV177A were commonly detected in OBI_B_ and OBI_C_ strains in both anti-HBs (+) and anti-HBs (−) populations.

## 4. Discussion

OBI is a worldwide health problem, and the reported prevalence significantly varies depending on locations, populations, and the sensitivity of diagnostic assays. The detection rate of OBI in blood donors was 1 : 1436 in our study, which was approximately two times lower than that in the data from South China reported by the National Institute of Diagnostics and Vaccine Development in Infectious Diseases of China (1 : 631) [12] but was higher than that in the data from Shenzhen Blood Center (1 : 3239) [4, 13]. Due to the similarity of the populations and diagnostic assays in these two studies, the differences in OBI rates may be due to the different prevalence rates of HBV infection in North and South China [14].

The distribution of HBV genotypes presents clear differences in geographical regions, ethnicities, and clinical outcomes [15]. Genotypes B and C are prevalent in Southeast Asia and China. In particular, genotype C circulates mainly in North China, Korea, Japan, and Thailand, and genotype B is predominant in South China and Southeast Asia [10, 16]. Several studies have shown the enhanced infectivity of genotype C, and genotype C is associated with more severe liver diseases, including cirrhosis and HCC [17, 18]. It was reported that HBV genotypes had a significant influence on the occurrence of OBI [5]. In our study, the mutation rates of pre-S1, pre-S2, and S in genotype C were higher than in genotype B, but no statistical differences were found. In addition, the mutation rates in S protein of anti-HBs (−) OBI were higher than anti-HBs (+) OBI samples (4.40% vs. 2.43% in genotype B, *P* > 0.05; 6.81% vs. 3.47% in genotype C, *P* < 0.05). Controversially, Wang JW et al. reported that the number and frequency of site substitutions in the S protein from anti-HBs (+) OBI_C_ strains were higher than those associated with anti-HBs (−) OBI_C_ strains, which suggested that the presence of anti-HBs might more effectively select variants in the S regions in genotype C than in genotype B OBI strains [4]. However, our results indicated that nonsynonymous mutations may occur randomly and were not selected by anti-HBs pressure. Further studies should be conducted based on larger samples and a strict control group to investigate whether the amino acid substitutions in HBV strains are significantly associated with clinical features and OBI occurrences in anti-HBs positive or negative patients.

Unlike HCV and some other hepatitis viruses, their antibodies could not provide protection and were the only evidence of infection. The anti-HBs were the neutralizing antibodies for HBV and the markers of recovery from acute infection or immunity from vaccination. The coexistence of HBsAg and anti-HBs seemed contradictory, but it was reported in many populations of different ages, sex, and immune situations [4, 6–8]. The mechanism behind anti-HBs (+) OBI is still unclear, and the research mainly focuses on the following aspects to explore it at present: (1) Pre-S/S gene. The pre-S/S gene encodes three envelope proteins named large S (encoded by regions of pre-S1, pre-S2, and S), middle S (encoded by regions of pre-S2 and S), and small S (encoded by only the S region) protein. The pre-S (pre-S1 and pre-S2) region contains several functional sites and is crucial for viral replication. The pre-S region also plays an essential role in interacting with the immune responses because it is rich in B/C cell epitopes [19]. The major hydrophilic region (MHR) is between aa100 and aa169 in the HBV S gene, and the “*α*” determinant is a relatively conserved region within MHR, which serves as the most important immunodominant region in all HBV strains and is essential to the detection of HBsAg and development of HBV vaccines [20]. Mutations or insertion or deletion in the HBV pre-S/S gene may affect the antigenicity, immunogenicity, expression, and secretion of HBsAg, leading to detection failure of HBsAg. It may also reduce or even abolish the virion's replication and/or secretion, exerting a negative effect on HBsAg presentation [21–24]. (2) The titer, types, or binding capacity of anti-HBs. Zhang ZH et al. found that the neutralization capacity of anti-HBs in OBI patients declined significantly, which indicated that the types or the binding capacity of anti-HBs in OBI patients might be different from that in vaccinators [25].

In this study, ps1A60V, ps1G73S, ps1V90A in pre-S1 and ps2I42T in pre-S2 regions were only detected in OBI_C_ strains obtained from blood donors with anti-HBs positive or negative. The functions of these mutations should be further studied. 13 individual substitutions in the sequences of the S region were found with high frequency (9.1∼45.5%) in OBI strains (Table 4). The mutations psN40S and psK122R were found in 27.3% and 45.5% of anti-HBs (−) OBI strains, respectively. The other 11 mutations were detected in anti-HBs(+) and anti-HBs(-) OBI populations. Regarding the distribution of the substitutions in genotypes of HBV, psN40S was uniquely observed in OBI_B_ carriers, 3 mutations were found in OBI_C_ strains, and the other 9 mutations were detected in both OBI_B_ and OBI_C_ strains. SK122I appeared to affect the biological properties of HBsAg and facilitated glycosylation of HBV [26], and sK122R was reported in OBI_B_ strains in both anti-HBs (+) and anti-HBs (−) OBI strains [4]. In our study, sK122R was uniquely found in anti-HBs (−) OBI strains, suggesting that sK122R may occur not due to anti-HBs pressure.

Several substitution sites were detected in this study, including sN40S, sY100S/C sK122R, sI126T/M, sQ129R/L, sM133T/L, sF134I/L/R, sK160R/S/K, sD144E/N/A, and sV177A, have been reported to be associated with OBI [19]. The classical immune escape mutations sG145A/K were found in both anti-HBs (+) and anti-HBs (−) OBI_C_ strains with low mutation frequency, suggesting that this classical mutation might be better detected due to the improvement in HBsAg detection reagents, and the mutation may occur randomly regardless of the pressure of anti-HBs.

## 5. Conclusion

The mutations in the pre-S/S region of OBI strains were analyzed and compared among different HBV genotype groups (OBI_B_ and OBI_C_) and serological groups (anti-HBs positive and anti-HBs negative). The amino acid substitutions occurred more frequently in OBI than in wt-HBV, providing possible explanations for the occurrence and existence of OBI. The amino acid mutation rate in the S protein of anti-HBs (−) OBI samples was higher than anti-HBs (+) OBI samples, suggesting that the mutations in the S region are not selected by anti-HBs pressure resulting from vaccination or a natural host immune response to HBV. Our study provided insights into the underlying mechanisms by which the OBI occurs and the anti-HBs present in OBI carriers. However, the numbers of OBI strains that could amplify the pre-S/S region were small, and all the samples came from Shaanxi. The source of HBV strains was relatively single. Further work should be conducted based on a multicenter and large sample size, and more accurate results could be obtained. Furthermore, functional analysis of relevant genetic variants should be performed to elucidate the mechanism of OBI with anti-HBs positive in the future.

## Figures and Tables

**Table 1:** The serological classification of HBsAg (−)/HBV DNA (+) samples.

Serological model	HBsAg	Anti-HBs	HBeAg	Anti-HBe	Anti-HBc	*N*	Rate (%)
1	−	−	−	−	−	10	10.53
2	−	+	−	−	−	7	7.37
3	−	+	−	−	+	24	25.26
4	−	+	−	+	+	3	3.16
5	−	−	−	+	+	5	5.26
6	−	−	−	−	+	46	48.42

HBsAg, hepatitis B surface antigen; anti-HBs, hepatitis B surface antibody; HBeAg, hepatitis B e antigen; anti-HBe, hepatitis B e antibody; anti-HBc, hepatitis B core antibody.

**Table 2:** The amino acids substitutions of envelope protein in OBI strains compared with wild-type HBV strains.

Region	Mutation frequency/strains	Mutation frequency/strains	*P* value
	(Mutation rate in OBI strains)	(Mutation rate in wt-HBV strains)	(OBI vs. wt-HBV)
*OBI*_*B*_			
pre-S1	5/4 (1.05%)	22/15 (1.23%)	1.000
pre-S2	7/4 (1.47%)	12/15 (0.67%)	0.095
S	52/10 (3.6%)	24/15 (1.11%)	<0.001

*OBI*_*C*_			
pre-S1	28/7 (3.36%)	22/15 (1.23%)	0.001
pre-S2	17/7 (2.04%)	9/15 (0.60%)	<0.001
S	79/11 (4.99%)	12/15 (0.56%)	<0.001

OBI_B_, OBI genotype B; OBI_C_, OBI genotype C; wt-HBV, wild-type HBV.

**Table 3:** The mutations in the pre-S/S region of OBI_B_ and OBI_C_ strains with anti-HBs positive or negative.

Protein	Lenth	Mutation	Genotype B	Genotype C	Sites common B/C
		Sites	Number of mutation	Number of mutation	Number of mutation
			Sites/frequency (mutation rate)	Sites/frequency (mutation rate)	Sites/frequency (mutation rate)
pre-S1	119	*anti-HBs*+/*anti-HBs*-	0/0	3/11 (1.32%)	2/4 (0.31%)
		*anti-HBs*+	3/3 (0.84%)	17/22 (3.70%)	
		*anti-HBs*-	2/2 (1.68%)	6/6 (2.52%)	

pre-S2	55	*anti-HBs*+/*anti-HBs*-	1/2 (0.91%)	2/6 (1.56%)	0/0
		*anti-HBs*+	6/6 (3.64%)	11/13 (4.73%)	
		*anti-HBs*-	1/1 (1.82%)	3/3 (2.73%)	

S	144	*anti-HBs*+/*anti-HBs*-	8/20 (1.39%)	10/37 (2.34%)	20/75 (2.48%)
		*anti-HBs*+	13/14 (2.43%)	20/30 (3.47%)	
		*anti-HBs*-	27/38 (4.40%)	31/49 (6.81%)	

pre-S/S	318	*anti-HBs*+/*anti-HBs*-	9/22 (1.03%)	15/54 (1.93%)	22/79 (1.60%)
		*anti-HBs*+	22/23 (2.09%)	48/65 (3.75%)	
		*anti-HBs*-	30/41 (3.95%)	40/58 (5.43%)	

OBI, occult hepatitis B virus infection; anti-HBs, hepatitis B surface antibody.

**Table 4:** High-frequency mutations of envelope protein in OBI strains classified with genotypes and the presence and absence of anti-HBs.

Region	Mutation	Frequency in anti-HBs (+) strains	Frequency in	*P*
			anti-HBs (−) strains	
OBI_C_				
pre-S1	A60V	2/8 (25%)	1/3 (33.3%)	1
	G73S	4/8 (50%)	1/3 (33.3%)	1
	V90A	2/8 (25%)	1/3 (33.3%)	1
pre-S2	I42T	3/8 (37.5%)	1/3 (33.3%)	1
S	T118R/V/M	3/10 (30%)	1/11 (9.1%)	0.311
	I126T/M	3/10 (30%)	2/11 (18.2%)	0.635
	D144E/N/A	1/10 (10%)	3/11 (27.3%)	0.586
OBI_B_				
S	N40S	0/10 (0%)	3/11 (27.3%)	0.214
OBI_B_/OBI_C_				
S	V/T47K/A/E	3/10 (30%)	4/11 (36.4%)	1
	K122R	0/10 (0%)	5/11 (45.5%)	0.035
	M133T/L	1/10 (10%)	3/11 (27.3%)	0.586
	F134I/L/R	1/10 (10%)	3/11 (27.3%)	0.586
	K160R/S/K	1/10 (10%)	5/11 (45.5%)	0.149
	V168A	1/10 (10%)	3/11 (27.3%)	0.586
	S174N	3/10 (30%)	3/11 (27.3%)	1
	L175S	2/10 (20%)	3/11 (27.3%)	1
	V177A	3/10 (30%)	2/11 (18.2%)	0.635

Anti-HBs, hepatitis B surface antibody; OBI_B_, OBI genotype B; OBI_C_, OBI genotype C.

## Data Availability

The data used to support the findings of this study are available from the corresponding author upon request.
